# Imaging and Treatment of Posttraumatic Ankle and Hindfoot Osteoarthritis

**DOI:** 10.3390/jcm10245848

**Published:** 2021-12-13

**Authors:** Tetyana Gorbachova, Yulia V. Melenevsky, L. Daniel Latt, Jennifer S. Weaver, Mihra S. Taljanovic

**Affiliations:** 1 Einstein Medical Center, Department of Radiology, Sidney Kimmel Medical College, Thomas Jefferson University, Philadelphia, PA 19141, USA; 2 Department of Radiology, UAB Medical Center, University of Alabama at Birmingham, Birmingham, AL 35249, USA; ymelenevsky@uabmc.edu; 3 Department of Orthopaedic Surgery, University of Arizona, Tucson, AZ 85724, USA; dlatt@ortho.arizona.edu; 4 Department of Radiology, University of New Mexico, Albuquerque, NM 87131, USA; jsweaver@salud.unm.edu; 5 Department of Radiology, University of New Mexico Health Sciences, Albuquerque, NM 87131, USA; mihrat@radiology.arizona.edu; 6 Departments of Medical Imaging and Orthopaedic Surgery, University of Arizona, Tucson, AZ 85724, USA

**Keywords:** ankle and hindfoot osteoarthritis, ankle arthroplasty, subtalar fusion, tibiotalarcalcaneal fusion

## Abstract

Posttraumatic osteoarthritis of the ankle and hindfoot is a common and frequently debilitating disorder. 70% to 90% of ankle osteoarthritis is related to prior trauma that encompasses a spectrum of disorders including fractures and ligamentous injuries that either disrupt the articular surface or result in instability of the joint. In addition to clinical evaluation, imaging plays a substantial role in the treatment planning of posttraumatic ankle and hindfoot osteoarthritis. Imaging evaluation must be tailored to specific clinical scenarios and includes weight bearing radiography that utilizes standard and specialty views, computed tomography which can be performed with a standard or a weight bearing technique, magnetic resonance imaging, and ultrasound evaluation. This review article aims to familiarize the reader with treatment rationale, to provide a brief review of surgical techniques and to illustrate expected imaging appearances of common operative procedures performed in the setting of posttraumatic ankle and hindfoot osteoarthritis, such as joint-preserving procedures, ankle fusion, subtalar fusion, tibiotalarcalcaneal fusion and ankle arthroplasty. Preoperative findings will be discussed along with the expected postoperative appearance of various procedures in order to improve detection of their complications on imaging and to provide optimal patient care.

## 1. Introduction

Osteoarthritis of the ankle and hindfoot is a common and frequently debilitating disorder. Although joint deterioration may result from a variety of disease processes, such as developmental, inflammatory and neoplastic conditions, 70% to 90% of ankle osteoarthritis is related to prior trauma [1,2]. Posttraumatic osteoarthritis (PTOA) of the ankle and hindfoot may be generated by a spectrum of disorders including fractures and ligamentous injuries that either disrupt the articular surface or result in instability of the joint. In addition to clinical evaluation, imaging plays a substantial role in the treatment planning of posttraumatic ankle and hindfoot osteoarthritis. This review article aims to familiarize the reader with treatment rationale, to provide a brief review of surgical techniques and to illustrate the expected imaging appearance of common operative procedures performed in the setting of the posttraumatic ankle and foot osteoarthritis, such as osteotomies, fusion procedures, and ankle arthroplasty.

## 2. Anatomic Considerations

The ankle or talocrural joint is a synovial joint formed by the trochlear surface of the talus, the inferior articular surface of the tibia, and the articular facets of the medial and lateral malleoli [3]. The bones are connected by a fibrous capsule lined by synovium and reinforced medially and laterally by collateral ligamentous complexes. The distal tibiofibular joint is a fibrous joint, united by the syndesmotic ligamentous complex that includes the interosseous ligament and the anterior and posterior tibiofibular ligaments. The subtalar joint, also referred to as the posterior subtalar or posterior talocalcaneal joint, is a synovium-lined articulation between the posterior calcaneal facet of the talus and the posterior talar facet of the calcaneus [3]. Ligamentous structures stabilizing the posterior subtalar joint include the talocalcaneal ligaments as well as components of the medial and lateral ligamentous complexes of the ankle, such as the superficial deltoid ligament medially and the calcaneofibular ligament laterally. The subtalar joint may communicate with the ankle joint in approximately 10 to 20% of persons [3,4]. This anatomic communication may provide a pathway for the spread of pathological processes, such as infection or inflammation, as well as therapeutic injections between these two joints. In addition, physiologic communication may exist between the ankle joint and tendon sheaths of the flexor hallucis longus or the flexor digitorum longus, or both in approximately 20% of patients. The anterior subtalar joint, also referred to as the talocalcaneonavicular joint, is separated from the posterior subtalar joint by the tarsal canal and sinus tarsi. The anterior subtalar joint is a synovium lined joint that encompasses articulation between the talar head and posterior surface of the navicular bone and articulations between the anterior and middle facets of the calcaneus and plantar aspect of the talar head [3]. Some variations in joint communications may exist, which may lead to a distinction of a separate middle subtalar joint.

## 3. Spectrum of Traumatic Disorders and Clinical Presentation

PTOA may result from cartilage damage sustained at the time of injury or chronic cartilage overloading that is caused by articular incongruity and instability [5]. Intraarticular fractures, such as tibial plafond fractures, may directly disrupt the articular surface, whereas extraarticular fractures may produce deformities resulting in abnormal joint mechanics, and avulsion fractures that involve supporting capsuloligamentous structures may lead to joint instability. In the absence of a fracture, a ligamentous disruption during ankle sprain may have a profound effect on the joint biomechanics causing malalignment and instability that lead to abnormal loading and rapid development of secondary degenerative arthritis. Classic studies demonstrated that even 1 mm of lateral displacement of the talus on the tibia reduces the tibiotalar contact area by 42 percent [6]. In addition, it has been shown that up to 95% of severe ankle sprains have concomitant chondral injuries [7]. Although chronic abnormal loading and residual incongruity contribute to joint degeneration, it is the extent of injury to the articular cartilage during initial trauma that is believed to be the major predisposing factor in the development of PTOA of the ankle [1]. PTOA of the subtalar joint may result from subtalar dislocations, talar and calcaneal fractures, and fracture-dislocations. Fundamentally, anatomic reconstruction of joint congruity is essential for functional recovery after intraarticular fractures; however, despite adequate management, subtalar arthritis may develop as a result of primary cartilage damage at the time of injury [6].

The onset of clinical symptoms of pain and dysfunction may occur years or decades after the original injury [1,8]. Patients present with ankle and hindfoot joint pain that is worse with activity or weight bearing, and instability, stiffness, or swelling that is initially relieved by rest. The onset of symptoms is usually insidious with slow progression over time. Ankle PTOA is a potentially debilitating disorder that may significantly impact a patient’s mobility and quality of life. Compared to osteoarthritis of other joints, patients with PTOA of the ankle are an average of 14 years younger at the time of diagnosis and progress more rapidly to end-stage disease, resulting in increased duration of pain and loss of function [1,8].

## 4. Imaging Evaluation

**Figure 1:**
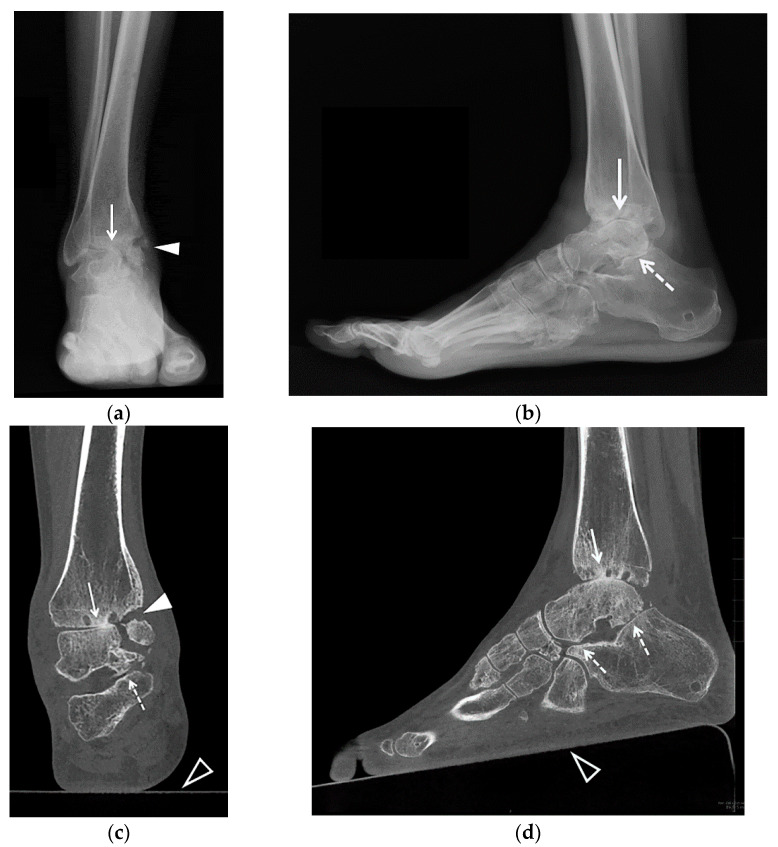
(**a**–**d**). A 25-year-old female with posttraumatic osteoarthritis of the right tibiotalar and subtalar joints. AP (**a**) and lateral (**b**) weight bearing radiographs and coronal (**c**) and sagittal (**d**) reformatted weight bearing CT images of the right ankle show an ununited medial malleolus fracture (arrowheads) and severe osteoarthritis of the tibiotalar (arrows) and subtalar joints (dashed arrows) with asymmetric joint space narrowing, subchondral sclerosis and small cyst-like changes. In (**b**) note the external fixator pin track in the calcaneal tuberosity. In (**c**,**d**) note a platform underneath of the foot (open arrowheads) with the CT images acquired in a cone-beam CT scanner dedicated to extremity imaging that allows the assessment of the alignment. Case courtesy of Imran Omar MD, Chicago, IL.

Imaging assessment of PTOA of the ankle and foot begins with radiography. Osteoarthritis is depicted by usually asymmetric joint space narrowing, osteophyte formation, subchondral bone sclerosis and cyst-like changes [9]. Chronic posttraumatic osseous deformities and malalignment may also be evident. Standard radiographic views include weight bearing anteroposterior (AP), mortise, and lateral views of the ankle [10]. Additional radiographic views are employed to visualize the posterior subtalar joint [11,12]. Broaden views are obtained to depict various parts of the subtalar joint on lateral projection using 10 degrees beam increments. The Harris-Beath view, or axial calcaneal projection, can demonstrate the posterior subtalar joint, the middle facets of the anterior subtalar joint and the sustentaculum, which allows assessment of calcaneal deformities in the axial plane. Hindfoot alignment evaluation is performed to detect varus or valgus malalignment and consists of either the long axial view or the hindfoot alignment view [13,14]. Compared to radiographs, computed tomography (CT) provides a superior assessment of the articular surfaces of the ankle joint and, in particular, the subtalar joint, depicting articular surface deformity, degenerative changes and joint incongruity [11]. Reformations in the coronal and sagittal planes can be reconstructed with different obliquities. Weight-bearing CT (WBCT) of the foot and ankle is an emerging technology that is increasingly being used by orthopedic surgeons for diagnostic and preoperative planning purposes. In contrast to standard CT scans, WBCT scans demonstrate the alignment of the bones and joints during loading and are superior in the characterization of posttraumatic deformities and instability [15] (Figure 1). Similar to CT, magnetic resonance imaging (MRI) provides multiplanar imaging that allows a better depiction of the complex anatomy of the ankle and subtalar joints. CT is also the study of choice for pre-operative planning for a total ankle arthroplasty. Scanning is obtained from the knee to the ankle and three-dimensional reconstructions are used to create patient specific instrumentation (PSI) for a total ankle arthroplasty. MRI also provides the unique ability to identify ligamentous injury, subchondral marrow abnormalities and cartilage lesions [16]. In the setting of osteoarthritis, ultrasound examination helps assess the joints for the presence of effusion and synovitis and can be used to target therapeutic intraarticular injections. It must be emphasized that radiographic evaluation remains the first and principal imaging modality in the setting of advanced osteoarthritis. Moreover, obtaining weight bearing radiographs is essential for both initial assessment and postoperative evaluation.

## 5. Treatment

### 5.1. Non-Operative Treatment

Currently, no effective treatments are available to prevent the progression of PTOA and evidence indicates that therapeutic interventions must occur early in order to modify the course of disease [1]. Nonoperative treatment of PTOA of the ankle includes bracing and intraarticular injections. Brace treatment of ankle arthritis is aimed at limiting motion and reducing axial loading [17]. Therapeutic joint injections most commonly are performed with corticosteroids or anesthetics [18]. Injections of the ankle and subtalar joints can be successfully performed under ultrasound or fluoroscopic guidance. Corticosteroids are used to provide short- to medium-term pain relief. The clinical duration of effect is considered to be inversely proportional to the solubility of the injected steroid. Intraarticular injections may serve both as therapeutic and diagnostic procedures [19,20]. Injection of local anesthetic, while giving very short-term pain relief, may assist in determining the patient’s source of pain in the presence of multiple confounding factors. In addition to confirming the placement of the needle during fluoroscopically guided joint injection, intraarticular administration of contrast may demonstrate the presence of communication between the ankle joint and the subtalar joint. Such communication results in medications being delivered to both joints and needs to be considered when interpreting symptom relief and when planning further surgical procedures, such as ankle fusion or subtalar fusion.

### 5.2. Operative Treatment

Several surgical options are available for patients with different stages of ankle PTOA and can be broadly categorized as joint-preserving and joint-nonpreserving procedures.

#### 5.2.1. Joint-Preserving Procedures

Joint-preserving procedures include arthroscopy or arthrotomy with debridement, distraction arthroplasty, osteochondral ankle joint resurfacing, and corrective osteotomies.

*Open or arthroscopic joint debridement* with removal of loose bodies, synovectomy, and resection of osteophytes may provide temporary relief of symptoms and is performed with variable success rates in patients with early osteoarthritis.

*Distraction arthroplasty*, or arthrodiastasis, is a technique where distraction force is applied to the joint for approximately 12–17 weeks using rigid external fixation by an Ilizarov apparatus or an anatomically located hinge to allow for a range of motion exercises. Current literature suggests that ankle joint distraction arthroplasty is a viable alternative treatment option in patients younger than 45 years with posttraumatic ankle osteoarthritis and preserved hindfoot motion. These procedures have been shown to reduce pain and improve mobility in patients with advanced osteoarthritis and delay the need for arthroplasty or fusion [21,22,23]. The therapeutic effect is considered to be due to the change in the metabolism of proteoglycans and the improvement of intra-articular inflammation [24]. Pain relief is generally incomplete. In patients with persistent pain and progressive ankle osteoarthritis, further therapeutic options can be later pursued [25].

*Supramalleolar osteotomy* is a realignment procedure reserved for eccentric cartilage loss secondary to excessive varus or valgus malalignment of the tibiotalar joint. Abnormal alignment results in focal increased pressure at the talar dome and tibial plafond, leading to asymmetric cartilage loss. Supramalleolar osteotomies are indicated for patients with at least 50% preservation of joint space [26]. The terms “closing” or “opening” in regard to osteotomies refer to either resecting or adding a wedge of bone, respectively, in order to correct the deformity.

**Figure 2:**
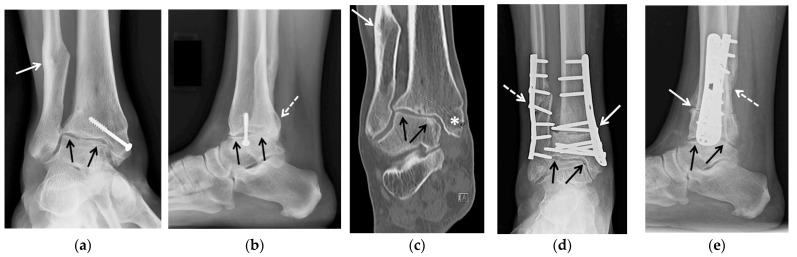
(**a**–**e**) A 47-year-old female with a history of remote trimalleolar right ankle fractures and posttraumatic tibiotalar joint osteoarthritis, treated with tibial wedge opening osteotomy and oblique fibular lengthening osteotomy. (**a**) Mortise and (**b**) lateral weight-bearing radiographs and (**c**) coronal reformatted CT image of the right ankle show a healed posttraumatic deformity of the medial malleolus with tibial valgus malunion transfixed by an interfragmentary screw (white asterisk in (**c**)) and healed posttraumatic deformities of the fibula (white arrow) and of the posterior malleolus (dashed white arrow). Note the asymmetric narrowing of the tibiotalar joint consistent with advanced posttraumatic osteoarthritis (black arrows). (**d**) Mortise and (**e**) lateral 3 months postoperative weight-bearing radiographs show improved alignment of the distal tibia (white arrow) and fibula (dashed white arrow) status post osteotomies with decreased tibiotalar joint space narrowing (black arrows). Osteotomy sites demonstrate partial union at 3 months, a normal finding.

Medial closing wedge osteotomy is used to correct valgus ankle deformity. A wedge-shaped fragment is resected from the medial distal tibial metadiaphysis, and stabilizing plate and screws are applied. Pre-existing shortening of the fibula due to malunion may affect tibiotalar joint alignment, necessitating a corrective lengthening osteotomy of the fibula [27] (Figure 2).

In patients with remaining valgus position of the calcaneus and abduction deformity of the mid- and forefoot, the deformity may be corrected by lateral lengthening calcaneal osteotomy [28].

Varus deformity of the tibiotalar joint can be corrected by medial opening wedge osteotomy or lateral closing wedge osteotomy. The medial opening wedge osteotomy is indicated in cases with a varus deformity less than 10° [26]. In patients with varus deformity of more than 10°, medial opening wedge osteotomy correction may be restricted by the fibula. This can be overcome by performing a lateral approach osteotomy of the fibula when a block of bone is removed. Subsequently, tibial lateral closing wedge osteotomy is performed [29].

#### 5.2.2. Arthrodesis

*Ankle arthrodesis* is a well-documented surgical treatment of end-stage ankle arthritis. It has been a preferred treatment of ankle arthritis because of its predictable outcomes. Besides PTOA, indications for primary ankle arthrodesis include osteonecrosis of the talus, symptomatic osteochondral lesions of the talus, neuroarthropathy, and failed total ankle arthroplasties. The goals of ankle arthrodesis are to decrease pain, improve function, and provide stability and alignment to allow the patients to return to their functional activity. When the pain originates within the ankle joint, a successful arthrodesis usually eliminates it, and pain relief is more reliable with fusion than with most other techniques. Short-term results and complication rates have been markedly improved by modern techniques of limited periosteal stripping, rigid internal fixation, and meticulous attention to alignment and position. Secondary operations, other than occasional hardware removals, are rarely needed [17]. The patient’s age, weight, compliance, comorbidities, and expectations are taken into account when considering arthrodesis.

Over 30 various ankle arthrodesis techniques have been developed since the first description by Albert in 1879. Ankle arthrodesis is commonly performed via an open approach, however, arthroscopic ankle arthrodesis, while limited to minimal angular deformities, has become increasingly popular during the past decade due to the advantage of minimizing significant soft tissue stripping. The classic open procedure described in 1948 involves resection of the distal fibula approximately 2 cm proximal to the ankle joint, which is used for autologous graft material [30]. Articular cartilage of the distal tibia and talar dome is removed to the vascular bone. The intervening gap is closed, ensuring the optimal plantigrade position of the foot. A fibular onlay graft is applied to the lateral aspect of the talus and tibia and stabilized by screws. The fibular graft can be split longitudinally and applied to both the anterior and lateral surfaces of the talus and tibia [31]. This technique has evolved for several decades and included the using lateral malleolus as a graft source, a change in the number of implants used in tibiotalar stabilization, and a variation in the direction of screw insertion. Additionally, anterior, posterior and medial transmaleollar approaches have been described [32]. Currently, an anatomic ankle fusion with fibular-sparing technique is widely utilized as it offers several advantages. An intact fibula allows increased surface area for union, preservation of the peroneal groove, prevention of valgus malalignment and lateral translation in cases of non-union and facilitates the possibility of conversion to a total ankle arthroplasty in the future [33]. The most frequent complications after tibiotalar and tibiotalocalcaneal arthrodesis involve nonunion, malunion, infection, and delayed wound healing. Additional complications include neurovascular injuries, adjacent hindfoot joint arthroses or laxity, malalignment, chronic edema, stress fractures, painful scars, and calluses [34].

**Figure 3:**
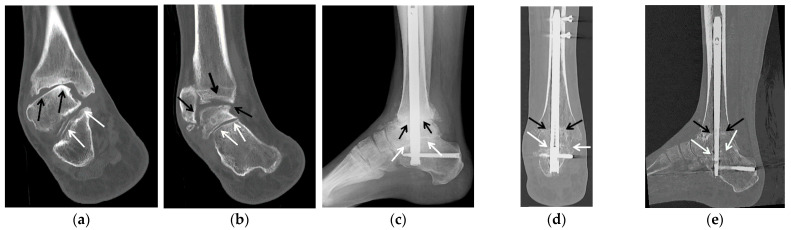
(**a**–**e**) A 58-year-old male with tibiotalar and subtalar osteoarthritis with varus alignment treated with tibiotalocalcaneal arthrodesis with intramedullary nail and iliac crest bone marrow aspirate. (**a**,**b**) Coronal reformatted preoperative CT images of the right ankle show advanced osteoarthritis of the tibiotalar (black arrows) and posterior subtalar (white arrows) joints with associated varus deformity. (**c**) Six weeks postoperative lateral weight-bearing radiograph shows a retrograde tibiotalocalcaneal intramedullary nail with interlocking screws in the calcaneus and distal tibial diaphysis transfixing the subtalar (white arrows) and tibiotalar (black arrows) joints with associated bone graft material. (**d**) Coronal and (**e**) sagittal reformatted CT images obtained 2.5 months after surgery show markedly improved alignment with progressive fusion across the subtalar (white arrows) and tibiotalar (black arrows) joints.

**Figure 4:**
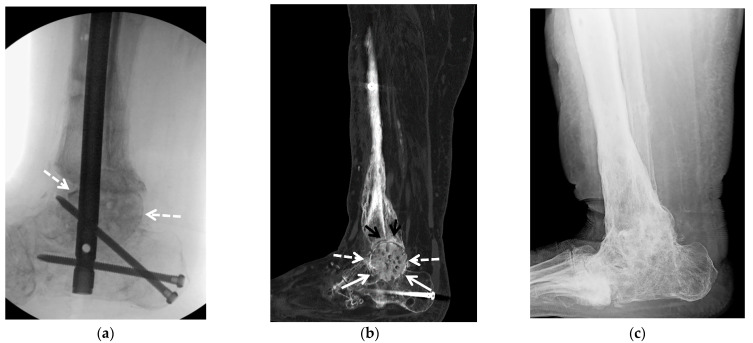
(**a**–**c**). A 58-year-old male with a complex medical history including diabetes mellitus, infected right Charcot midfoot and hindfoot as well as advanced secondary osteoarthritis, treated with staged fusion: first external fixation with antibiotic spacer and intravenous antibiotics followed by tibiotalocalcaneal fusion with a structural femoral head allograft. (**a**) Fluoroscopic lateral intraoperative image of the right ankle shows a retrograde tibiotalocalcaneal intramedullary nail with two distal screws in the calcaneus and across the posterior calcaneal facet, and a structural femoral head allograft placed at the talar dome bone void and resection site (white arrows). (**b**) Sagittal reformatted CT image obtained 3.5 months after surgery shows the structural femoral head allograft (dashed white arrows) replacing the talar dome, between the calcaneus (white arrows), talar head and tibial plafond (black arrows). Partially visualized is tibiotalocalcaneal fixation hardware. (**c**) Seven months postoperative lateral weight-bearing radiograph status post hardware removal shows complete solid bony fusion across the subtalar and tibiotalar joints with complete incorporation of the structural femoral head allograft.

*Tibiotalocalcaneal arthrodesis* is an effective salvage procedure used for the treatment of conditions that affect both the ankle and subtalar joints. Indications include failed tibiotalar arthrodesis, extensive osteonecrosis of the talus, failed total ankle arthroplasty, Charcot arthropathy, and gross instability presenting as flail ankle. This type of fusion is usually accomplished by retrograde intramedullary nail placement [35] (Figure 3). Compression at the site of arthrodesis may be lost due to bone resorption or settling. In order to improve healing by maintaining compression at the fusion sites, new techniques, such as the placement of internal pseudoelastic elements, have been implemented [36]. Limb shortening due to structural bone loss in tibiotalocalcaneal arthrodesis can negatively impact the patient’s gait and weight-bearing. Structural bone deficit of the talus and ankle and hindfoot malalignment, often seen in end-stage degenerative joint disease, present complex reconstruction challenges that may necessitate the use of structural allograft [37]. A structural femoral head allograft (FHA) can be successfully used to maintain the height of the limb and correct the deformity during tibiotalar fusion [38] (Figure 4).

**Figure 5:**
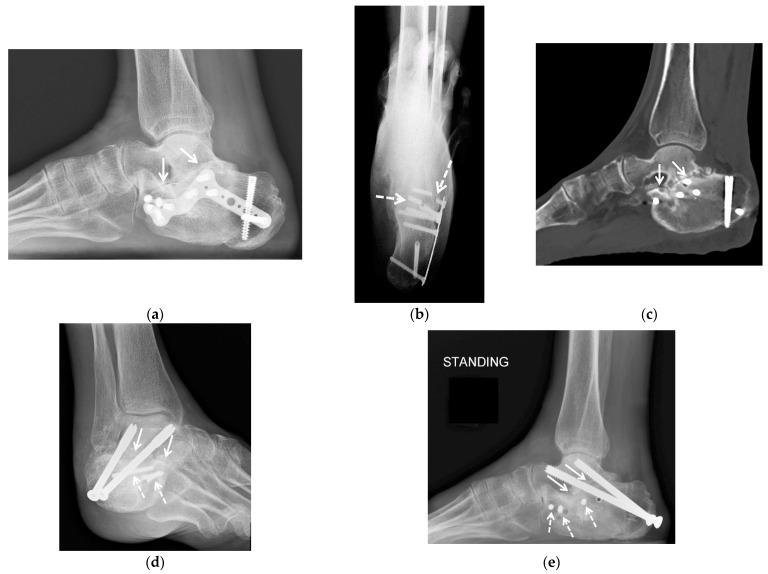
(**a**–**c**) A 67-year-old female with a history of remote comminuted right calcaneus fracture treated with plate and screws fixation with subsequent hardware fracture and subtalar osteoarthritis with subfibular impingement treated with calcaneal hardware removal, calcaneal exosteoectomy and subtalar arthrodesis with cellular allograft. (**a**) Lateral weight-bearing radiograph of the right foot shows a healed calcaneus fracture status post plate and screws fixation. Note marked asymmetric narrowing of the subtalar joint consistent with advanced posttraumatic osteoarthritis (white arrows). (**b**) On the axial Harris weight-bearing radiograph note fracture of multiple fixation screws (dashed white arrows). (**c**) Sagittal reformatted CT image shows advanced posttraumatic osteoarthritis of the subtalar joint with asymmetric joint space narrowing, scattered subchondral sclerosis and cyst-like changes. (**d**) Broden and (**e**) lateral radiographs obtained 2.5 months after subtalar arthrodesis show interval removal of the calcaneal plate and multiple screws with three broken screw fragments remaining (white dashed arrows). Note two retrograde partially threaded cannulated screws transfixing the subtalar joint (white arrows) with early fusion about the fixation screws.

**Figure 6:**
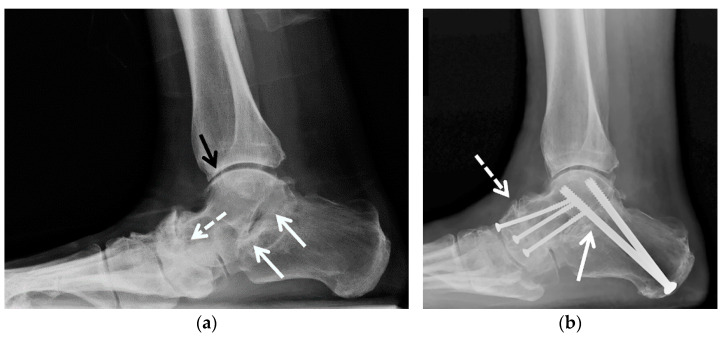
(**a**,**b**) A 72-year-old male with pes planus and severe talonavicular greater than subtalar osteoarthritis was treated with double hindfoot arthrodesis. (**a**) Preoperative weight-bearing lateral radiograph of the right foot shows severe osteoarthritis of the talonavicular (dashed white arrow) greater than subtalar joints (white arrows) and mild osteoarthritis of the tibiotalar joint (black arrow). (**b**) Weight-bearing lateral radiograph obtained 3 weeks after surgery shows three smaller partially threaded cannulated screws transfixing the talonavicular joint (dashed white arrow) and two larger, partially threaded cannulated screws transfixing the posterior subtalar joint (white arrow). Note the significant improvement of hindfoot alignment.

*Subtalar arthrodesis* may be performed as a primary procedure for subtalar arthrosis, usually secondary to calcaneal fracture, rheumatoid arthritis, primary osteoarthritis, or a non-resectable talocalcaneal coalition. Advantages of this procedure include preservation of hindfoot motion and lower risk of arthritis in adjacent joints [39]. During the procedure, cartilage and subchondral bone are removed from the talar and calcaneal articular surfaces; hindfoot alignment can be corrected by using wedge resection or graft application. One or two large caliber cannulated screws are placed to traverse the talocalcaneal articulations (Figure 5). Subtalar fusion may be paired with talonavicular fusion in a so-called double hindfoot arthrodesis. This procedure leaves the calcaneofibular joint free which is believed to act as a “force-dissipating’’ factor during ambulation [38] (Figure 6).

**Figure 7:**
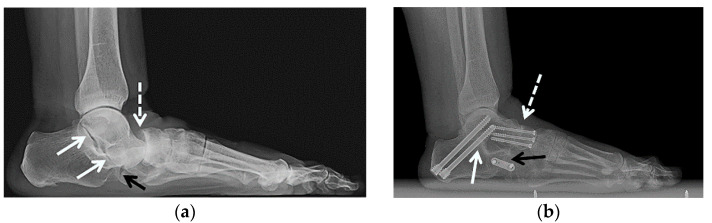
(**a**,**b**) A 64-year-old female with a history of traumatic right talonavicular dislocation several months prior presented with continued pain, pes planus with plantar valgus and equinus contracture and posttraumatic osteoarthritis, subsequently treated with triple arthrodesis. (**a**) Lateral weight-bearing radiograph of the right foot shows per planus with dorsal subluxation of the talonavicular joint and superimposed advanced osteoarthritis (dashed white arrow). Note the associated mild asymmetric narrowing of the subtalar (white arrows) and calcaneocuboid joints (black arrow). (**b**) Postoperative weight-bearing lateral radiograph shows interval triple arthrodesis with two Herbert screws transfixing the posterior subtalar joint (white arrow), three partially threaded cannulated screws transfixing the talonavicular joint (dashed white arrow) and a short plate with two screws transfixing the calcaneocuboid joint (black arrow) with solid bony bridging across the arthrodesis sites.

*Triple arthrodesis* is a fusion of the talocalcaneal, talonavicular, and calcaneocuboid joints (Figure 7). This procedure is reserved for cases when available conservative measures have failed, and a more limited surgical procedure will not provide appropriate pain relief and reduction of the deformity. The indications for triple arthrodesis include severe subtalar, talonavicular and calcaneocuboid degenerative disease, sequelae of talar neck fractures with subtalar joint involvement, posttraumatic hindfoot instability, and non-resectable calcaneonavicular or talocalcaneal coalition. Triple arthrodesis can be used to treat painful deformities due to inflammatory arthritides, neurogenic, and neuromuscular disorders. Triple arthrodesis is used for the treatment of fixed hindfoot deformity in the setting of adult-acquired flatfoot deformity (AAFFD, stage 3) [40]. The subtalar joint is fixated through the use of either one or two partially threaded cancellous screws. The talonavicular and calcaneocuboid joints are fixated using a combination of screws, staples, or plates [41].

Complications can occur with these various surgical procedures, as with any operative intervention, including edema, hematoma, seroma, dehiscence, ulceration, infection, and nerve damage. Specific complications of the osteotomy procedures and arthrodesis include nonunion, delayed union, malunion, and graft failure. With successful arthrodesis procedures in the long term, when movement at the joint is eliminated, accelerated secondary osteoarthritis of the neighboring joints often develops because of increased motion [39,42]. General principles of postoperative imaging evaluation include assessment of position and hardware integrity, graft incorporation, the adequate fusion of the joint with gradual obliteration of the joint space, presence of bone loss and zones of avascular sclerotic bone. All of these parameters are principally evaluated with serial weight bearing radiography and additionally with CT. Nuclear medicine studies including single photon emission computed tomography (SPECT) scan with technetium and Indium-111 tagged white blood cells serve to determine the extent and activity of nonunion, infection, and osteonecrosis. In cases of suspected infection, a bone biopsy for cultures may be needed before a definitive procedure.

#### 5.2.3. Ankle Arthroplasty

**Figure 8:**
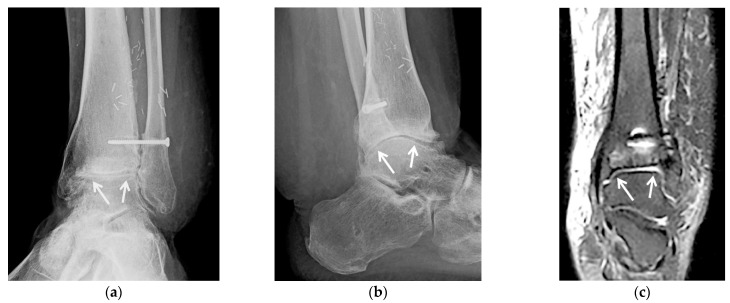

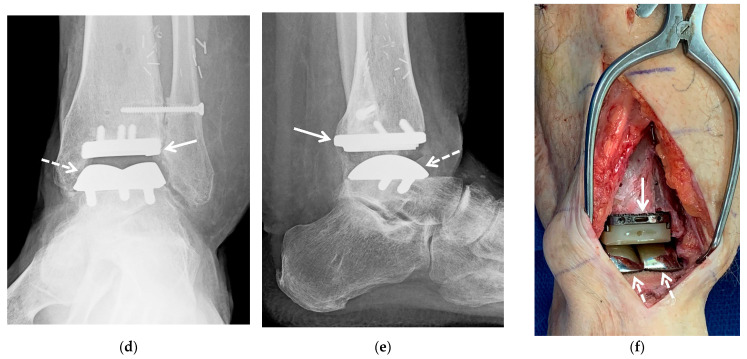
(**a**–**f**) A 36-year-old male with posttraumatic osteoarthritis of the left tibiotalar joint and equinus contracture resulting from open distal tibia fracture treated with open reduction internal fixation and free flap, with previous hardware removal, subsequently treated with ankle arthroplasty and gastrocnemius resection. (**a**) Mortise and (**b**) lateral weight-bearing radiographs of the left ankle show marked asymmetric narrowing of the tibiotalar joint (white arrows) with marginal osteophytes and intraarticular bodies consistent with severe osteoarthritis. Note tracts in the calcaneus and talus related to removed surgical hardware and retained laterally placed screw transfixing the distal tibiofibular syndesmosis. Numerous vascular clips overly the soft tissues of the lower leg. (**c**) Coronal fluid sensitive MR image shows asymmetric narrowing of the tibiotalar joint (white arrows). (**d**) Mortise and (**e**) lateral postoperative radiographs show interval placement of total ankle arthroplasty (Wright Medical Infinity with an Inbone talus). Note well seated tibial (white arrow) and talar (dashed white arrow) prosthesis components. (**f**) The intraoperative image shows tibial (white arrow) and talar components (dashed white arrow) in place.

**Figure 9:**
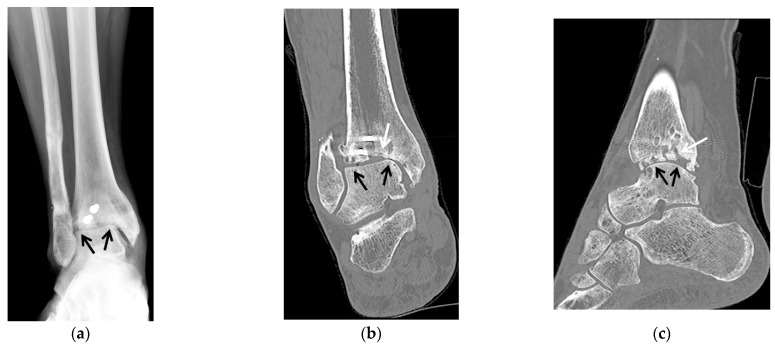

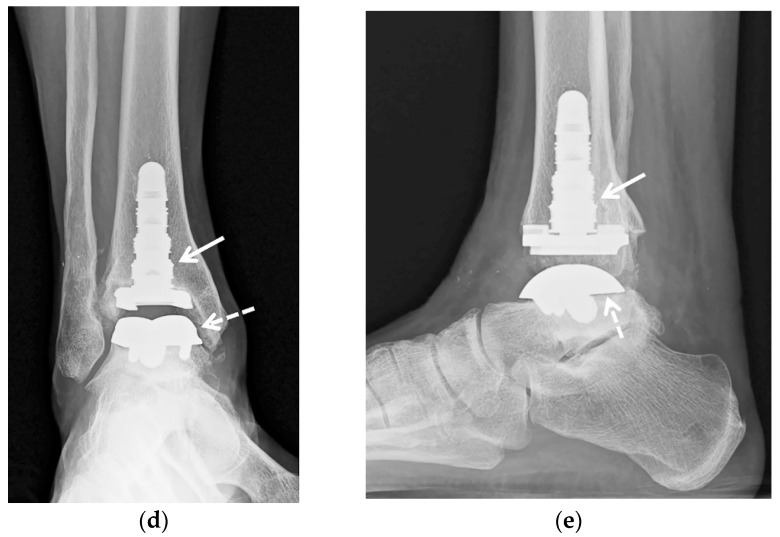
(**a**–**e**) A 57-year-old female with a history of remote open reduction and surgical fixation of the right ankle fractures, revision posterior malleolus 2 years prior and posttraumatic osteoarthritis with 2 cm devitalized bone at the central tibial plafond; patient subsequently treated with hardware removal and total ankle arthroplasty (Wright Medical with Inbone tibia and talus). (**a**) Mortise radiograph and (**b**) coronal and (**c**) sagittal reformatted CT images of the right ankle show asymmetric narrowing of the tibiotalar joint (black arrows) consistent with advanced osteoarthritis. In (**a**,**b**) note two retained screws in the distal tibia. In (**b**,**c**) note marked irregularity of the tibial plafond with scattered foci of sclerosis corresponding to devitalized bone (white arrow). (**d**) Mortise and (**e**) lateral postoperative weight-bearing radiographs show total ankle arthroplasty with well seated tibial (white arrow) and talar components (white arrowhead).

Traditionally, ankle arthrodesis has been a primary surgical option for patients with PTOA who have failed conservative treatment. However, ankle arthrodesis produces gait dysfunction and increased stress on the neighboring joints that frequently leads to accelerated osteoarthritis in these joints. Total ankle arthroplasty (TAA) represents an alternative to arthrodesis offering an advantage of preservation or improvement of mobility of the tibiotalar joint while reducing pain. Historically, ankle arthroplasty has not been as successful as replacement of other joints and early TAA designs had unacceptably high rates of failure. Newer generation designs combined with improved surgical technique and level of training among the surgeons have demonstrated substantially improved results that lead to a gradual increase in the utilization of TAA in the treatment of PTOA [43,44,45]. However, the rates of complications and failure with TAA remain greater than those seen in total knee and hip arthroplasty [46]. The most common indication for TAA is the treatment of advanced ankle arthropathy, failed prior ankle surgery, and ankle arthropathy with prior hindfoot or midfoot fusion for whom preserved functional range of motion is desired [47]. Appropriate patient selection is very important for a good clinical outcome of ankle arthroplasty [45,48]. Ideally, TAA is to be performed in middle-aged to older patients without significant comorbidities, with normal to low body mass index (BMI), adequate tibial and talar bone stock, stable and well-aligned hindfoot, and no lower extremity neurovascular impairment [45]. In the past decade indications for TAA have expanded and now include the presence of correctable deformities and less restrictive age and BMI criteria. TAA is contraindicated in the presence of active bone or soft tissue infection, neurovascular compromise, inadequate soft tissue support and poor bone stock. Designs of ankle prostheses have evolved towards improving osteointegration, decreasing the extent of bony resection and decreasing the degree of constraint allowing rotation and sliding motions in addition to flexion and extension (Figure 8). Two general categories of TAA implants are currently in use: two-component design, also referred to as “fixed bearing” (Figure 9), and three-component design, referred to as “mobile bearing” [43,49]. A polyethylene (PE) spacer is locked to the tibial base plate in a two-component prosthesis and is freely mobile in a three-component design. Prostheses are further distinguished by the composition and configuration of the metallic component, and the surgical approach for insertion, which may necessitate fibular osteotomy and syndesmotic fixation.

The overall complication rate in TAA has been reported as high as 20%. The most common reason for implant failure is aseptic loosening, followed by persistent pain and periprosthetic infection [48]. Overall TAA complications may be categorized based on several parameters, such as level of risk of development of implant failure or based on the time of occurrence [50,51]. Depending on the time since surgery, complications may be categorized as intraoperative, early postoperative, or delayed [47,51]. Early postoperative complications include infection, delayed wound healing, medial malleolar stress fracture and distal tibial or fibular fractures. Late complications consist of periprosthetic fracture, aseptic loosening, expansile osteolysis, impingement, polyethylene spacer wear and migration, subsidence, syndesmotic nonunion, heterotopic ossification, osteoarthritis in neighboring joints, and chronic regional pain syndrome. Imaging evaluation of TAA includes several modalities, such as radiography, CT, MRI, and nuclear medicine studies, with serial weight bearing radiographs being the mainstay of the imaging follow-up. Imaging surveillance may detect early findings of aseptic loosening or infection by demonstrating subtle abnormalities, such as subsidence, early osteolysis, and angular deformities [47].

## 6. Conclusions

Posttraumatic osteoarthritis of the ankle and hindfoot is a common and frequently debilitating disorder that compromises quality of life. Several non-operative treatments and surgical options may be considered with respect to the individual patient’s therapy goals. In addition to clinical evaluation, imaging plays important role in treatment planning, monitoring treatment results and early diagnosis of surgical complications. Weight bearing radiographs are essential for both initial assessment and postoperative evaluation.
